# Practical Notes on Diagnosis and Treatment

**Published:** 1910-07-23

**Authors:** 


### Practical Notes on Diagnosis and Treatment.

**Rickets.**

A cold or tepid daily sponge with salt water prevents cold-taking and is a valuable help to metabolism. It is especially necessary owing to the sweating.—*Dr. E. Holt.*

**The Balsams and Nephritis.**

It should be borne in mind that the inunction of storax and balsam of Peru in the treatment of scabies is liable to produce albuminuria and even acute nephritis.—*Dr. John Allan.*

**Cardiac Murmurs in Infants.**

If under two years old, murmurs mean congenital defects if they are loud; if soft they are generally functional. Acquired organic heart disease is very rare under three years of age.—*Dr. J. Abercrombie.*

**Caffeine.**

This is not only a diuretic, but also a valuable heart tonic. One of its chief uses is in the slow labouring heart of senility, as it somewhat accelerates the heart's action and invigorates the systole.—*Dr. Seymour Taylor.*

**Functional Heart Disease.**

In women "angina pectoris" is nearly always functional. Patients with functional derangements of the heart often make them an excuse for avoiding exercise and for taking stimulants. If any cardiac symptom can be walked off it may be put down as functional.—*Sir Wm. Broadbent.*

**Pain in Hæmorrhoids.**

Uncomplicated piles are never the cause of acute pain. Some special complication must be searched for in every case in which a patient complains of acute suffering. The common causes of such pain are thrombosis, prolapse, strangulation, and fissure.—*Mr. Pearce Gould.*

**Strychnine and the Heart.**

This popular remedy has no special effect on the heart, in spite of the properties which have been ascribed to it from clinical evidence alone. It has been recommended in exactly opposite conditions on evidence that speaks more for unreasoning faith in the drug than for the beneficial properties of the drug itself.—*Dr. James Mackenzie.*

**Rheumatoid Arthritis.**

The drugs I have found most useful in the treatment of rheumatoid arthritis are guaiacol and potassium iodide.—*Dr. A. P. Luff.*

**Taxis in Strangulated Hernia.**

I would strongly enjoin on you that early operation promises the greatest success. I strongly hold to the view that a really strangulated hernia is rarely reducible by taxis.—*Mr. J. W. Hulke.*

**Insomnia.**

If a man cannot get to sleep for a long time and then wakes up with a start, this generally means overwork; a gouty man goes off to sleep till about 2 a.m., when he wakes and cannot get off again, while he is generally full of depressing thoughts.—*Dr. Mitchell Bruce.*

**Hæmorrhage after Tonsillotomy.**

Make a mass with a little water of 3 parts of tannic acid and 1 part of gallic acid. Rub a small piece of this firmly over the bleeding surface, making counter-pressure with the other hand on the side of the head. I have never known this to fail.—*Mr. Mark Hovell.*

**Osteo-arthritis.**

It is a great mistake to look upon the prognosis in rheumatoid arthritis as a hopeless one, and to accept the dictum that once it has attacked a joint the affection will steadily spread till the patient is a hopeless cripple; such cases do occur, but they are the exception, just as galloping consumption is the exception.—*Dr. W. Calwell.*

**Infant Feeding.**

In many of the starch-free foods there is an excess of carbohydrates which interferes with the assimilation of fat, even if this be present in sufficient quantity. Ill-effects may be delayed and the apparent suitability of the food leads to its continuance and favours the commencement of rickets. Before trying condensed milk or patent foods, always add sodium citrate to the diluted cow's milk. The anti-scorbutic property of fresh milk is an important point and one not to be lightly dispensed with.—*Dr. Still.*

